# Synthesis of rGO/CoFe_2_O_4_ Composite and Its Magnetorheological Characteristics

**DOI:** 10.3390/ma17081859

**Published:** 2024-04-18

**Authors:** Yang Lv, Chengjie Gong, Yuzhen Dong, Hyoung Jin Choi

**Affiliations:** 1 School of Materials Science and Engineering, Harbin Institute of Technology Weihai, 2 West Wenhua Road, Weihai 264209, China; 2021211612@stu.hit.edu.cn (Y.L.); 2021211658@stu.hit.edu.cn (C.G.); 2 Department of Polymer Science and Engineering, Inha University, Incheon 22212, Republic of Korea

**Keywords:** rGO, CoFe_2_O_4_, composite, magnetorheological, yield stress

## Abstract

In this study, composite particles of rGO/CoFe_2_O_4_ were synthesized using a solvothermal method to fabricate a low-density magnetorheological (MR) material with enhanced sedimentation stability. The morphology and crystallographic features of rGO/CoFe_2_O_4_ were characterized via SEM, TEM, and XRD, and its magnetic properties were tested using VSM. The MR fluid was formulated by blending rGO/CoFe_2_O_4_ particles into silicone oil. Under different magnet strengths (*H*), a rotational rheometer was used to test its MR properties. Typical MR properties were observed, including shear stress, viscosity, storage/loss modulus, and dynamic yield stress ($\tau_{dy}$) following the Herschel–Bulkley model reaching 200 Pa when *H* is 342 kA/m. Furthermore, the yield stress of the MR fluid follows a power law relation as *H* increases and the index changes from 2.0 (in the low *H* region) to 1.5 (in the high *H* region). Finally, its MR efficiency was calculated to be about 10^4^% at *H* of 342 kA/m.

## 1. Introduction

Magnetorheological (MR) fluids are usually suspensions of magnetic particles distributed in a base liquid [1]. As a class of magnetically responsive smart materials, MR fluids are widely valued for their unique MR effect [2,3]. Upon applying or removing an external magnetic field (*H*), MR fluids can convert between a liquid and a solid-like state within milliseconds [4]. When *H* is applied, the magnetic particles within MR fluids are polarized. The magnetic dipoles interact and organize into chain-like structures along the *H* direction. These chain-like structures resist flow and increase viscosity, making MR fluids a solid-like state [5]. When *H* is removed, MR fluids return to a liquid state. The characteristics of MR fluids find application across various engineering domains, such as dampers or shock absorbers [2], polishing [6], vehicle suspension [7,8], biomedical applications [9], soft robots [10], MR electrolytes in batteries [11], and so on. Moreover, the millisecond response time of MR fluids makes them one of the most rapid electromechanical interfaces [12]. Carbonyl iron (CI) particles are extensively utilized when preparing MR fluids due to their high saturation magnetization, ease of synthesis, and other favorable properties [13]. Nevertheless, MR fluids utilizing CI particles face a notable problem of magnetic particle sedimentation, which causes a considerable reduction in the MR effect [12,13,14]. Cobalt ferrite (CoFe_2_O_4_) is a cubic spinel-structured ferrite widely studied for its excellent electromagnetic properties and application characteristics in electronic products [15]. Meanwhile, CoFe_2_O_4_, with low density and excellent magnetic characteristics, has also attracted widespread attention for its MR performance [16,17].

Graphene, a two-dimensional (2D) material, has been extensively researched for its excellent electronic transport properties [18,19,20], mechanical properties [21,22], thermal conductivity [23], optical transparency [24], and other properties [25]. These properties enable graphene to have broad application prospects in important fields, including medicine, sustainable energy, composite materials, and so on [26,27,28]. A highly utilized graphene production approach is employing graphene oxide (GO) as a precursor, followed by removing oxygen-based functional groups from the GO surface through thermal or chemical reduction methods [29,30]. GO can be obtained by oxidation of natural graphite. This process introduces reactive oxygen-based functional groups, including hydroxy, carboxyl, epoxide, and carbonyl groups, onto the basal planes and borders of the graphene-derived layers, increasing the distance between layers [31,32]. In particular, it has been widely noted that these reactive functional groups are helpful when synthesizing GO-based composite materials, such as the synthesis of RGO-Fe_3_O_4_ composite [33], graphene oxide/polyethylene glycol composite [34], Cellulose–Graphite Oxide Composite [35], and so on.

A nanocomposite consisting of CoFe_2_O_4_ nanoparticles and rGO, characterized by outstanding magnetic properties, a large specific surface area, and low density, can enhance the MR fluid’s sedimentation stability and is expected to demonstrate typical MR behavior. This study utilized a modified Hummer’s method [36] to synthesize GO. In order to avoid the difficulty of dispersion in silicone oil caused by oxygen-based functional groups in GO, GO was reduced to reduced graphene oxide (rGO) [37]. Finally, the rGO/CoFe_2_O_4_ composite synthesized via a solvothermal method was dispersed into silicone oil to study its MR characteristics under different *H*.

## 2. Materials and Methods

### 2.1. Synthesis of Graphene Oxide (GO)

The synthesis of GO followed a modified Hummer’s method [36]. A quantity of 2 g of sodium nitrate (NaNO_3_) and graphite (4 g) were dispersed in 280 mL sulfuric acid (H_2_SO_4_) to obtain a homogeneous suspension with intense stirring. Then 12 g of potassium permanganate (KMnO_4_) was introduced into the suspension, followed by 30 min of stirring and 15 min of sonication. The obtained mixture was slowly poured into 700 g of water in a fume hood, and then a hydrogen peroxide (H_2_O_2_) solution (200 g of H_2_O_2_ dissolved in 400 water) was introduced. The reaction solution was slowly stirred for 20 min, and centrifugation was used to wash the product with water until the pH was constant. Finally, the resulting GO block was dispersed in water, peeled off into GO sheets via sonication for 4 h, and freeze-dried.

### 2.2. Fabrication of Reduced GO/Cobalt Ferrite (rGO/CoFe_2_O_4_)

GO (0.5 g) was dispersed in H_2_O (150 mL) following 1.5 h of sonication while stirring. Next, Fe(NO_3_)_3_ (6.875 g) and Co(NO_3_)_2_ (2.475 g) were introduced, and the mixture’s pH was adjusted to 10. The reaction solution was moved to an autoclave and subjected to heating at 180 °C. After an 18 h reaction, it was cooled to room temperature. Then a water wash was performed with magnetic separation. Finally, the drying process was carried out.

### 2.3. Preparation of MR Fluid

The MR fluid was formulated by blending 10% volume of rGO/CoFe_2_O_4_ particles into 1000 cSt silicone oil. The MR fluid was then subjected to shaking (VORTEX Genius 3, IKA, Staufen, Germany) and sonication (Powersonic 410, Hwashin, Seoul, Republic of Korea) to get a uniform suspension. In order to obtain uniformly dispersed MR fluid, sufficient shaking and ultrasonic treatment were performed using a vortex mixer (VORTEX Genius 3, IKA, Germany) and ultrasonic processor (Powersonic 410, Hwashin, Seoul, Republic of Korea).

## 3. Results and Discussion

### 3.1. Characterization of Synthesized Materials

**Figure 1:**
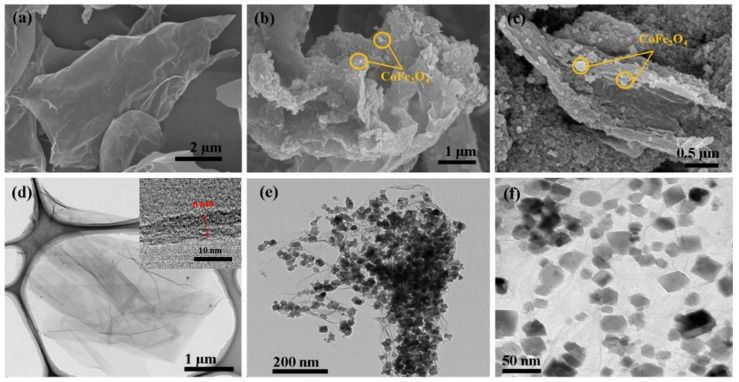
SEM images of (**a**) GO sheet, (**b**,**c**) rGO/CoFe_2_O_4_, and TEM images of (**d**) GO sheet, (**e**,**f**) rGO/CoFe_2_O_4_ composite. Inset in (**d**) shows side view of GO sheet.

Figure 1a,d depict the GO sheet’s SEM (S-430, Hitachi, Tokyo, Japan) and TEM (CMM-220, Phillips, Boston, MA, USA) images, respectively.

The GO sheet’s surface appears smooth, and the inset in Figure 1d depicts that the GO sheet’s thickness is approximately 6 nm. It is noteworthy that the graphite appears wrinkled after oxidation. This wrinkling phenomenon is due to the oxidation process promoting the introduction of oxygen-containing functional groups, thereby transforming the sp^2^ (planar structure) to sp^3^ (tetrahedral structure) hybridization [38]. Figure 1b,c,e,f depict the rGO/CoFe_2_O_4_ composite’s SEM and TEM images, respectively. The oxygen-based functional groups on the surface of GO result in a high density of negative charges on its surface. Cobalt and iron cations are electrostatically attracted and adsorbed onto the GO surface. Subsequently, CoFe_2_O_4_ nanoparticles are formed and anchored onto the GO surface via the solvothermal reaction. Meanwhile, during the solvothermal reaction, GO reduction occurs, resulting in the final rGO/CoFe_2_O_4_ composite material. As observed, the rGO/CoFe_2_O_4_ composite’s surface exhibits a rougher appearance compared to GO, and the CoFe_2_O_4_ particles are evenly distributed on the rGO without aggregation. The presence of rGO effectively disperses CoFe_2_O_4_ particles and avoids agglomeration, increasing the composite’s specific surface area [10,39]. In addition, the density of the rGO/CoFe_2_O_4_ composite was tested to be 4.05 g/cm^3^ with a gas pycnometer (AccuPyc 1330, micromeritics), notably lower than that of the pure phase CoFe_2_O_4_ particles (5.29 g/cm^3^) [40], indicating that the rGO/CoFe_2_O_4_ composite has a considerable advantage in solving the magnetic particle sedimentation problem in MR fluids.

**Figure 2:**
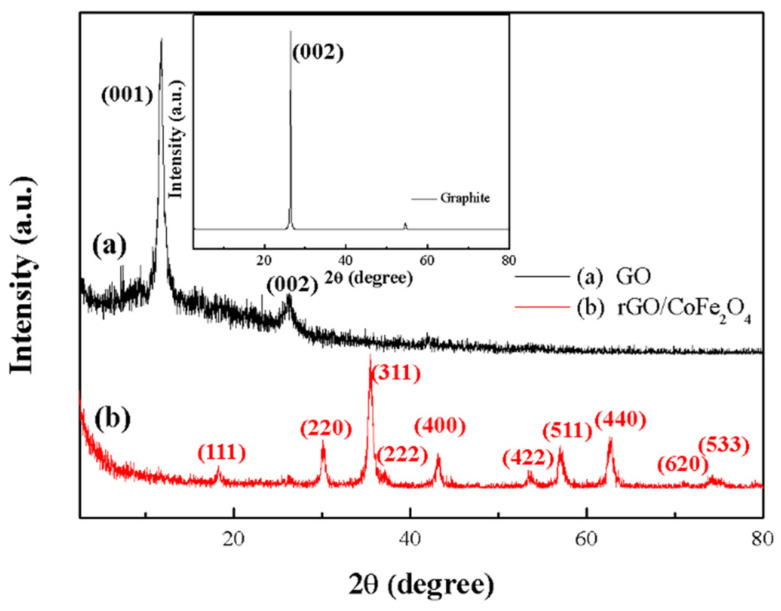
XRD patterns of (**a**) GO and (**b**) rGO/CoFe_2_O_4_. Inset depicts XRD pattern of graphite.

In Figure 2, the inset reveals that the graphite’s XRD (DMAX-2500, Rigaku, Tokyo, Japan) diffraction peak is at 2θ = 26.4°, indicating the (002) lattice plane and a 0.336 nm inter-layer distance.

The sharp diffraction peak indicates a high level of crystallization in the graphite. According to Figure 2a, the diffraction peak of GO is located at 2θ = 10.4°, indicating the (001) lattice plane and a 0.850 nm inter-layer distance. The sharp peak position of GO (001) shifts to the left relative to graphite (002), and the inter-layer distance increases. The increase in inter-layer distance is caused by the introduction of oxygen-containing functional groups during the oxidation of graphite. This indicates that graphite is transformed into GO by the oxidation process. GO exhibits a broad peak at 2θ = 26.4° due to the process efficiency that results in a small amount of graphite not being wholly converted [41]. According to Figure 2b, the characteristic peaks of the CoFe_2_O_4_ are distinctly observable at 2θ = 18.2°, 29.8°, 35.3°, 37.0°, 42.9°, 53.3°, 56.4°, 61.2°, 70.7°, and 74.0°, which correspond to the lattice planes (111), (220), (311), (222), (400), (422), (511), (440), (620), (533). These diffraction peaks match well with the JCPDS data (#221086) [42]. The positions of the three strongest peaks are 29.8° (220), 35.3° (311), and 61.2° (440), and the space group is Fd-3m (No. 227) [43], which proves that CoFe_2_O_4_ belongs to the spinel structure. Notably, no (001) GO diffraction peak exists in the rGO/CoFe_2_O_4_ XRD patterns, which indicates an efficient reduction of GO to rGO [44]. The XRD analysis confirms the successful fabrication of the rGO/CoFe_2_O_4_ composite.

**Figure 3:**
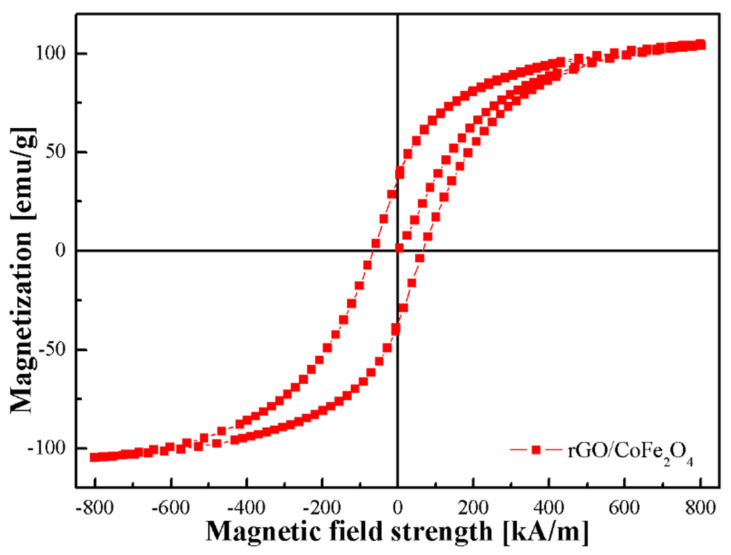
Magnetization curve of rGO/CoFe_2_O_4_ composite.

Figure 3 depicts the magnetization curve recorded during the vibrating sample magnetometry (VSM, Lake Shore Cryotronics, Westerville, OH, USA) test of the rGO/CoFe_2_O_4_ composite at 300 K, with *H* ranging from −800 kA/m to 800 kA/m.

The composite has a remanence ($M_{r}$) of 34.9 emu/g and a coercivity of 67.7 kA/m (850.7 Oe), which cannot be ignored. Notably, the saturation magnetization ($M_{s}$) of the rGO/CoFe_2_O_4_ composite measures 104.6 emu/g, surpassing the typical value of pure CoFe_2_O_4_ of 74.08 emu/g [15]. On the one hand, the higher $M_{s}$ can be explained by the larger particle size of the synthesized CoFe_2_O_4_ particles [45,46,47,48]. On the other hand, compared to pure CoFe_2_O_4_, the composite of rGO/CoFe_2_O_4_ affects the super exchange interaction, consequently influencing $M_{s}$ [49]. This high $M_{s}$ value is highly desirable because it contributes to enhanced yield strength and rapid response for MR fluids.

### 3.2. Magnetorheological Effect

#### 3.2.1. Shear Stress and Shear Viscosity

**Figure 4:**
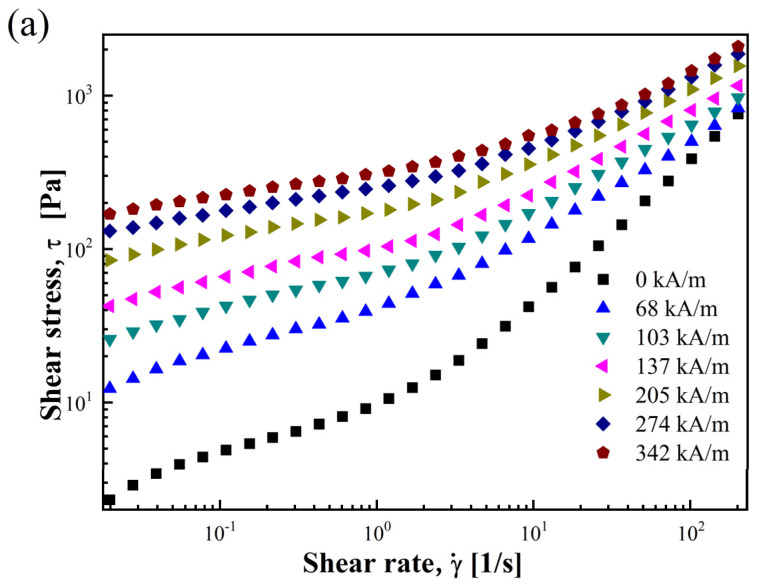

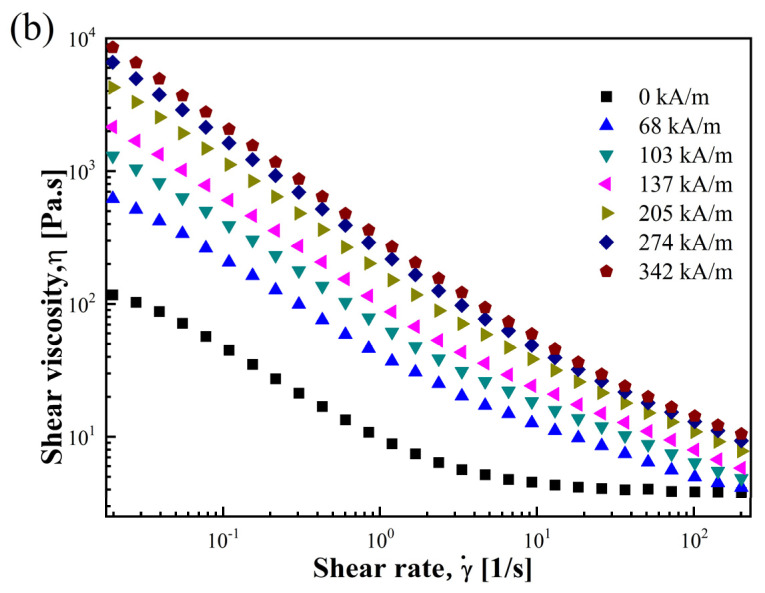
Flow curve for rGO/CoFe_2_O_4_-based MR fluid under various *H* (**a**) shear stress and (**b**) shear viscosity curves as a function of shear rate.

The rGO/CoFe_2_O_4_-based MR fluid’s MR properties were tested with a rotational rheometer (MCR 300, Anton-Paar, Stuttgart, Germany). The shear rate ($\dot{\gamma }$) was set across a range of 0.01 to 200 1/s, and *H* in the tests were set to 0, 68, 103, 137, 205, 274, and 342 kA/m. Figure 4a is the log–log graph indicating the relationship between shear stress (τ) and $\dot{\gamma }$ of the rGO/CoFe_2_O_4_-based MR fluid at different *H.*

When *H* is applied, the τ of the MR fluid is higher than when *H* = 0. At the same $\dot{\gamma }$, the higher the *H*, the higher the τ. At low $\dot{\gamma }$, the MR fluid at all six *H (H* ≠ 0) exhibited high τ, which increased slowly with increasing $\dot{\gamma }$. This is attributed to the strong chain-like structures formed by magnetic dipole–dipole interactions between the rGO/CoFe_2_O_4_ particles, which are resistant to disruption caused by the increasing $\dot{\gamma }$ [50]. When $\dot{\gamma }$ is high, the MR fluid’s chain-like structure is gradually disrupted, leading to a faster increase in τ with increasing $\dot{\gamma }$.

Figure 4b shows the shear viscosity (η) for rGO/CoFe_2_O_4_-based MR fluid as a function of $\dot{\gamma }$. It can be observed that η decreases with increasing $\dot{\gamma }$ at different *H*, suggesting evident shear-thinning behavior. In addition, η significantly increases after the input of *H* because of the alignment of the rGO/CoFe_2_O_4_ particles within the MR fluid along the direction of *H*, forming a chain-like structure. When *H* increases, the chain-like structure becomes more stable, enhancing resistance to breaking under shear and contributing to a higher η of the MR fluid. However, as $\dot{\gamma }$ increases, the MR fluid’s internal structure gradually breaks down, causing a decrease in η and showing the shear-thinning characteristic. When $\dot{\gamma }$ is high enough, the change in η is insignificant due to the complete collapse of the chain-like structure.

#### 3.2.2. Storage/Loss Modulus and Relaxation Modulus

**Figure 5:**
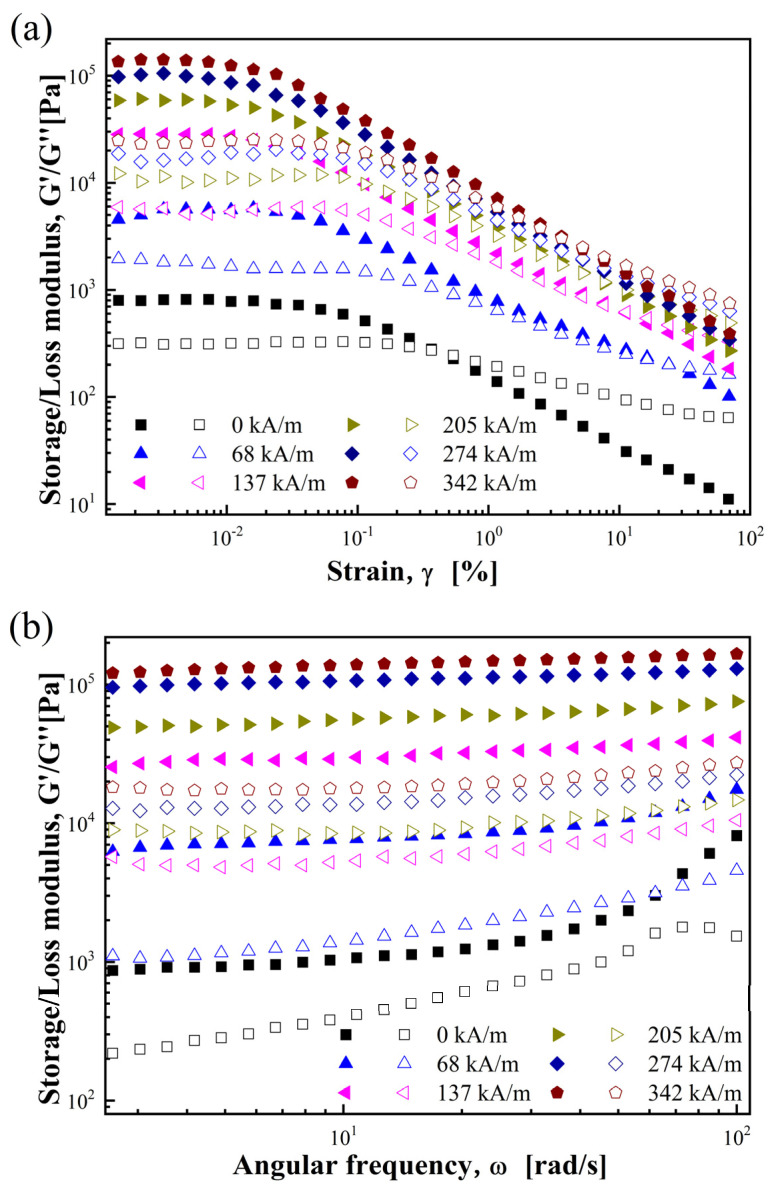
(**a**) Strain and (**b**) frequency dependence of storage (closed) and loss (open) modulus for rGO/CoFe_2_O_4_-based MR fluid.

To further investigate the rGO/CoFe_2_O_4_-based MR fluid’s viscoelastic behavior, different *H* (0–342 kA/m) were selected for oscillation tests at a steady 6.28 rad/s frequency. To examine how storage modulus (G′) and loss modulus (G″) change in response to strain (γ), γ was set to increase in the range of 0.001–100%. Figure 5a depicts the variation of G′ and G″ versus γ for the rGO/CoFe_2_O_4_-based MR fluid.

With an increase in γ, both G′ and G″ exhibit a plateau region over a smaller range of γ, recognized as the linear viscoelastic (LVE) region [51], where the G′ and G″ remain unaffected by γ. In the LVE region, G′ of the rGO/CoFe_2_O_4_-based MR fluid surpasses G″, indicating the dominance of its elastic properties and a quasi-solid state. Once γ of the rGO/CoFe_2_O_4_-based MR fluid exceeds the critical strain of 0.01% here, G′ and G″ decrease quickly. When γ surpasses the critical strain, due to the high γ, the chain-like structure within the MR fluid gets disrupted, making the MR fluid change from quasi-solid to fluid. In response to this transition, there is an irreversible decrease in both G′ and G″, and G′ even surpasses G″ when γ is high.

In the LVE region, a constant γ of 0.01% was applied for frequency sweep tests within the angular frequency (ω) range of 1–100 rad/s. Figure 5b shows the changes of G′ and G″ with ω for the rGO/CoFe_2_O_4_-based MR fluid. Upon applying *H*, G′ and G″ exhibit a plateau region, showing that the MR fluid’s internal structure makes it exhibit clear solid-like characteristics rather than liquid ones. The increase in G′ due to the increase in *H* indicates that the stronger *H* leads to enhanced interparticle interactions, and the MR fluid exhibits a stronger solid-like behavior. Moreover, at a specific *H*, G′ is consistently higher than G″ over a wide ω range, indicating that the rGO/CoFe_2_O_4_-based MR fluid is mainly characterized by its elastic properties rather than its viscous properties [52].

The time-dependent shear relaxation modulus (G(t)) of the rGO/CoFe_2_O_4_-based MR fluid can be calculated using the Schwarzl equation [53] as follows:

(1) $$G\left(t\right)≅G^{\prime }\left(\omega \right)-0.560G^{\prime \prime }\left(\omega /2\right)+0.200G^{\prime \prime }\left(\omega \right)$$

**Figure 6:**
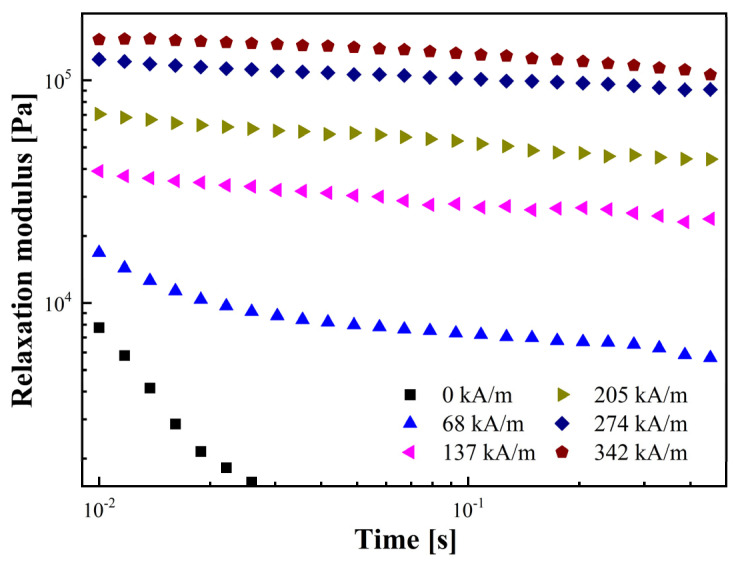
Relaxation modulus calculated from the storage and loss modulus as a function of time.

This equation can overcome the limits of mechanical tests and predict the MR fluid’s ultrafast relaxation behavior. Figure 6 depicts G(t) in relation to time for the rGO/CoFe_2_O_4_-based MR fluid.

The evident decrease in G(t) when *H* = 0 indicates the MR fluid’s liquid-like behavior. However, the G(t) of the MR fluid demonstrates a plateau state upon application of *H*, indicating quasi-solid behavior and no stress relaxation in the MR fluid.

#### 3.2.3. Dynamic and Elastic Yield Stress

To effectively consider the impact of $\dot{\gamma }$ on τ, the Herschel–Bulkley model was employed for fitting. When $\dot{\gamma }$ approaches zero, τ can be approximated as the dynamic yield stress ($\tau_{dy}$), as represented in the following equation [54]:

(2) $$\tau =\tau_{dy}+K{\dot{\gamma }}^{n}$$

where K is the consistency index; n is the flow behavior index; and $\dot{\gamma }$ is the shear rate.

Table 1 presents the Herschel–Bulkley model’s fitting parameters for $\tau_{dy}$, K, and n under various *H*.

**Figure 7:**
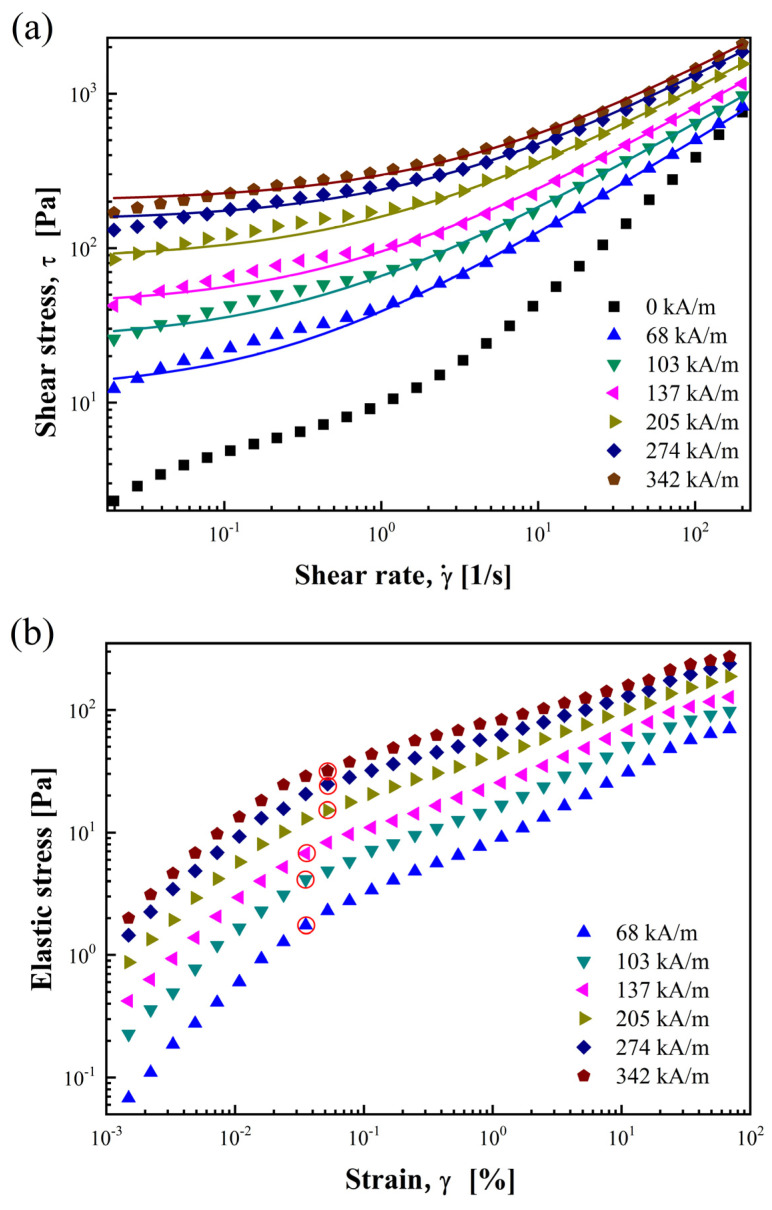
(**a**) Shear stress as a function of shear rate and (**b**) elastic stress as a function of strain under various *H*.

Notably, all fitted parameters for n are less than 1, indicating shear-thinning behavior in the MR fluid, which aligns with the conclusion drawn from Figure 4b. The solid lines shown in Figure 7a were fitted based on the data from Table 1.

The comparison between the fitting lines and experimental data in Figure 7a demonstrates a high level of agreement, suggesting that the Herschel–Bulkley model effectively fits the rGO/CoFe_2_O_4_-based MR fluid.

Elastic stress ($\tau_{e}$) can be determined by applying the following formula to the dynamic oscillatory strain amplitude sweep data:

(3) $$\tau_{e}=G^{\prime }\cdot \gamma$$

Figure 7b depicts the functional relationship between $\tau_{e}$ and γ for the rGO/CoFe_2_O_4_-based MR fluid at various *H*. Each turning point on the slope of the curve, as indicated by a red circle in the figure, is called the elastic yield point, corresponding to the elastic yield stress ($\tau_{ey}$) at a certain *H*. On the left side of the elastic yield point, the $\tau_{e}$ of the rGO/CoFe_2_O_4_-based MR fluid exhibits a linear increase with the rise of γ. However, the rate of increase in $\tau_{e}$ notably decelerates on the right side of the elastic yield point.

**Figure 8:**
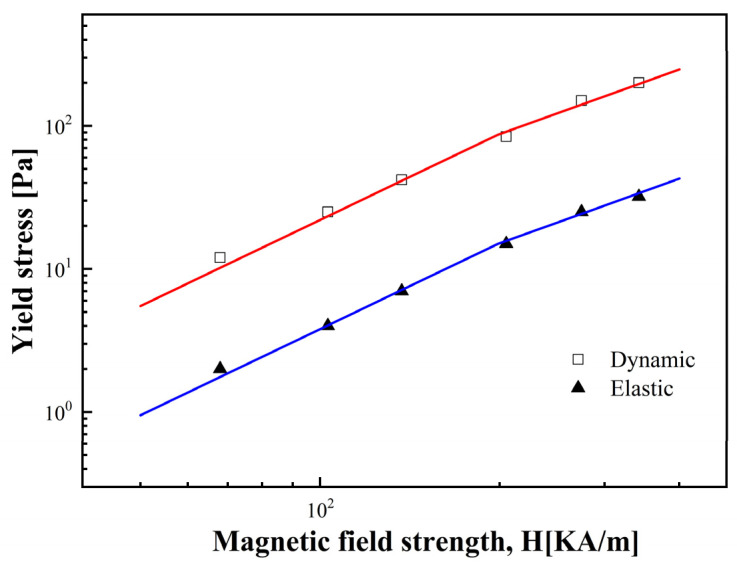
Dynamic yield stress (cubic) and elastic yield stress (trigonal) as function of *H*.

Figure 8 shows how $\tau_{dy}$ and $\tau_{ey}$ change with *H*. Typically, yield stress ($\tau_{y}$) and *H* have a power law relationship as follows:

(4) $$\tau_{y}\propto H^{m}$$

In the MR fluid, $\tau_{y}$ rises as *H* increases. When *H* is low, due to the magnetic polarization of particles, $\tau_{y}$ is directly proportional to $H^{2}$, following [55], as follows:

(5) $$\tau_{y}\propto \phi \mu_{o}H^{2}$$

where ϕ represents the MR fluid’s volume fraction, and $\mu_{o}$ is the vacuum magnetic permeability. When *H* increases, the chain-like structure is less affected by *H*, and local magnetization saturation dominates $\tau_{y}$, which can be represented as follows:

(6) $$\tau_{y}=\sqrt{6}\phi \mu_{0}M_{s}^{1/2}H^{3/2}$$

where $M_{s}$ represents the saturation magnetization.

From Figure 8, it is evident that as *H* increases, there exists a critical magnetic field strength ($H_{c}$) where the fitted curve’s slope shifts from 2.0 to 1.5. Due to the presence of $H_{c}$, the relationship between $\tau_{y}$ and *H* can be expressed using the following general equation [56]:

(7) $$\tau_{y}\left(H_{0}\right)=\alpha H_{0}^{2}\left(\frac{\tanh\sqrt{H_{0}/H_{C}}}{\sqrt{H_{0}/H_{C}}}\right)$$

where α depends on the MR fluid’s susceptibility, ϕ, and other physical constants. There are two distinct limiting relations between $\tau_{y}$ and $H_{0}$, as follows:

(8) $$\tau_{y}=\alpha H_{0}^{2}{\;\;\;\;H}_{0}\ll H_{c}$$

(9) $$\tau_{y}=\alpha \sqrt{H_{c}}H_{0}^{3/2}\;\;H_{0}\gg H_{c}$$

Equations (8) and (9) indicate that in the case of a low value of *H*, magnetic particles within the MR fluid are mutually attracted, leading to the creation of chain-like structures.

When *H* exceeds $H_{c}$, the MR fluid gradually becomes saturated, and $\tau_{y}$ is less affected by *H*. As depicted in Figure 8, the fitted curve’s slope for $\tau_{y}$ as a function of *H* undergoes a transition as *H* reaches $H_{c}$, demonstrating a high level of agreement with the experimental data.

**Figure 9:**
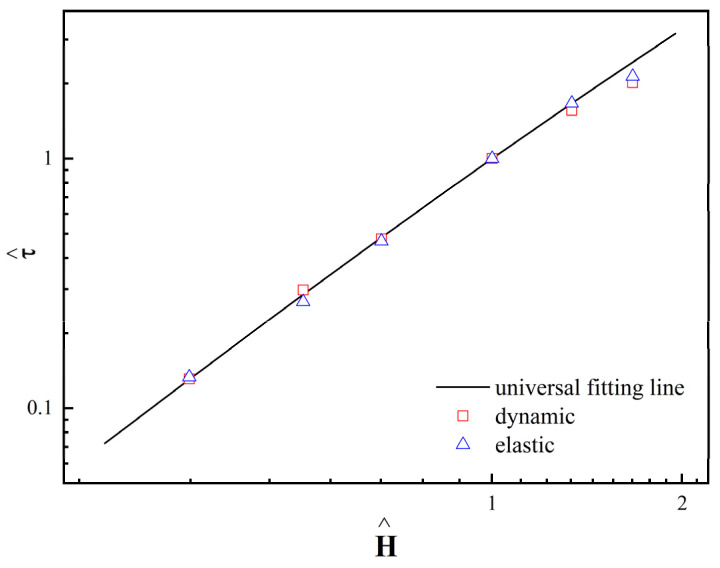
Dynamic and elastic yield stress as a function of *H* and universal fitting line.

According to Equation (7), when $H_{0}=H_{c}$, we can obtain $\tau_{y}\left(H_{0}\right)=\alpha H_{0}^{2}tanh1$, which simplifies to $\tau_{y}\left(H_{0}\right)=0.762\alpha H_{0}^{2}$. From this, we can obtain the following relationship:

(10) $$\hat{\tau }=1.313{\hat{H}}^{3/2}\tanh\sqrt{\hat{H}}$$

where $\hat{\tau }=\tau_{H_{0}}/\tau_{H_{c}}$, $\hat{H}=H_{0}/H_{C}$. As depicted in Figure 9, Equation (10) allows the data to be integrated into a unified curve.

#### 3.2.4. MR Efficiency

**Figure 10:**
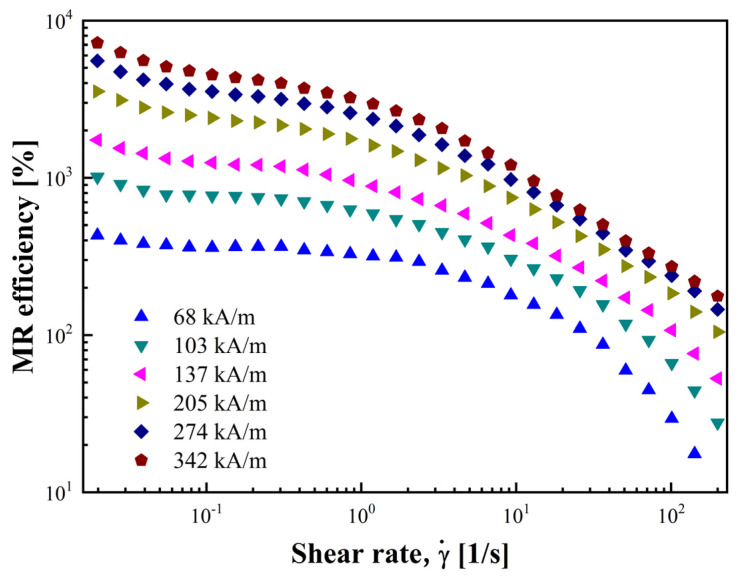
MR efficiency as a function of shear rate under various *H*.

MR efficiency quantifies the viscosity gain that can be achieved by the MR fluid at a specific *H* and $\dot{\gamma }$, which is critical for MR fluids operating in a flow state. The MR efficiency can be obtained by converting the data of the viscosity curve with the following formula:

(11) $$\mathrm{MR}\;\mathrm{efficiency}\;=\frac{\eta_{H}-\eta_{0}}{\eta_{0}}\times 100\%$$

where $\eta_{H}$ and $\eta_{0}$ represent η of the MR fluid at *H* and 0, respectively. Figure 10 depicts the MR efficiency as a function of $\dot{\gamma }$ and *H*.

The graph illustrates a decrease in MR efficiency with an increase in $\dot{\gamma }$. This decrease is due to the gradual disruption of chain-like structures as $\dot{\gamma }$ increases, reducing their resistance to the flow regime. In addition, at a constant $\dot{\gamma }$, increasing *H* enhances MR efficiency by forming a stronger internal structure that is more rigid to external shear [57]. MR efficiency is essential in engineering, and for MR devices such as dampers that need to operate in a flow state, the yield stress metric is no longer appropriate [58].

## 4. Conclusions

This work synthesized the rGO/CoFe_2_O_4_ composite using a solvothermal method. The synthesized GO and rGO/CoFe_2_O_4_ were characterized using SEM, TEM, and XRD, confirming the successful combination of rGO with CoFe_2_O_4_ particles. The VSM testing shows an expected high $M_{s}$ for the rGO/CoFe_2_O_4_ composite. The flow curves demonstrated the rGO/CoFe_2_O_4_-based MR fluid’s typical MR behaviors. G′ and G″ for the MR fluid obtained from oscillation tests reveal its viscoelastic behavior. G(t) was calculated with a Schwarzl equation. For the rGO/CoFe_2_O_4_-based MR fluid, τ and $\tau_{dy}$ conformed well to the Herschel–Bulkley model. Furthermore, $\tau_{y}$ was found to be proportional to $H^{2}$ when $H\ll H_{c}$, while it was proportional to $H^{3/2}$ when $H\gg H_{c}$. Finally, the rGO/CoFe_2_O_4_-based MR fluid’s MR efficiency was calculated, providing guidance for its potential applications in the flow state. To summarize, the rGO/CoFe_2_O_4_-based MR fluid can achieve a reversible transition between liquid-like and solid-like states. By applying different magnetic fields, we can control its rheological properties (τ, η, G′ and G″). These parameters are the basis for controlling system variables in engineering applications, such as valves, dampers, vehicle suspensions, and engine mounts.

## Figures and Tables

**Table 1:** Fitting parameters of Herschel–Bulkley model.

*H*/(kA/m)	$\tau_{dy}$	K	n
68	12	27	0.78
103	25	41	0.63
137	42	53	0.59
205	84	76	0.58
274	150	89	0.56
342	200	97	0.56

## Data Availability

Data are contained within the article.
